# Noise resilient leaky integrate-and-fire neurons based on multi-domain spintronic devices

**DOI:** 10.1038/s41598-022-12555-0

**Published:** 2022-05-19

**Authors:** Cheng Wang, Chankyu Lee, Kaushik Roy

**Affiliations:** Department of Electrical and Computer Engineering, Purdue University, West Lafayette, 47907 IN USA

**Keywords:** Nanoscale devices, Electrical and electronic engineering, Computational science, Neural circuits

## Abstract

The capability of emulating neural functionalities efficiently in hardware is crucial for building neuromorphic computing systems. While various types of neuro-mimetic devices have been investigated, it remains challenging to provide a compact device that can emulate spiking neurons. In this work, we propose a non-volatile spin-based device for efficiently emulating a leaky integrate-and-fire neuron. By incorporating an exchange-coupled composite free layer in spin-orbit torque magnetic tunnel junctions, multi-domain magnetization switching dynamics is exploited to realize gradual accumulation of membrane potential for a leaky integrate-and-fire neuron with compact footprints. The proposed device offers significantly improved scalability compared with previously proposed spin-based neuro-mimetic implementations while exhibiting high energy efficiency and good controllability. Moreover, the proposed neuron device exhibits a varying leak constant and a varying membrane resistance that are both dependent on the magnitude of the membrane potential. Interestingly, we demonstrate that such device-inspired dynamic behaviors can be incorporated to construct more robust spiking neural network models, and find improved resiliency against various types of noise injection scenarios. The proposed spintronic neuro-mimetic devices may potentially open up exciting opportunities for the development of efficient and robust neuro-inspired computational hardware.

## Introduction

The significant advancements of deep artificial neural networks (ANNs) in various domains of artificial intelligence (AI) such as image classification^1^, autonomous driving^2^, and natural language processing^3^ are accompanied by an exponential increase in computational complexity. The high computational requirements from data-intensive AI algorithms have placed a pressing challenge for developing efficient AI hardware. At present, while ANNs have demonstrated human-level performance on various cognitive tasks^4^, the power consumption of most AI algorithms implemented in state-of-the-art hardware is still substantially higher than that of a human brain. Inspired by the ultra-high efficiency and robustness of brains, neuromorphic computing aims to emulate behaviors of biological systems in order to achieve high efficiency at cognitive processing. Spiking neural networks (SNNs), which incorporate temporal dynamics of spikes as inspired by biological neural systems, has become an emerging paradigm that could potentially provide efficient and reliable platforms for AI processing^5–7^.

In order to construct efficient spike-based computational systems, it is imperative to provide compact and energy-efficient hardware emulation of key building blocks such as neurons and synapses. However, implementing bio-plausible neuronal functionalities based on conventional CMOS circuits typically desires a large number of interconnected transistors, which can be both area expensive and power hungry^8^. Emerging beyond-CMOS technologies, such as various non-volatile memories (NVM), are well-positioned for realizing neuromorphic building blocks efficiently with compact footprints. Prototypes of spiking neurons have been recently demonstrated exploiting various NVM technologies such as Ag-oxide memristor^9^ and phase change material^10^, ferroelectric field-effect transistor(FeFET)^11^, as well as spintronics^12, 13^. Among the various approaches based on emerging technologies, spintronic implementations provide the highest endurance thanks to the absence of ion motions in the writing process^14^. Since the state of neurons are updated frequently during both inference and training, the inherent high endurance of spintronics makes it particularly appealing for emulating neurons. While implementations of binary or stochastic neurons can be directly achieved exploiting the bidirectional polarization of magnetization^13^, mimicking analog-valued functionality (such as the accumulation of membrane potential in leaky integrate-and-fire neurons) remains challenging using spin-based technologies. Magnetic domain wall motions and skyrmions have been proposed to provide continuous modulation of states in spintronic devices, but large footprints (1 μm) and special designs of device geometry are required^12, 15^. In addition, the motions of domain walls and skyrmions are unpredictable and difficult to control due to strong sensitivity to shape/defect-related local pinning in fabricated devices, leading to undesirable device variability and repeatability issues that can significantly hinder large-scale system implementations^16^.

In this work, we propose a new spintronic device model to provide hardware emulation of leaky integrate-and-fire (LIF) neurons by exploiting the magnetization dynamics of multi-granular structures. Leveraging the significant advances of magnetic storage medium over the past few decades, magnetic granular structures are capable of sustaining multiple domains in devices with critical dimensions well below 100 nm^17, 18^. By incorporating an exchange-coupled composite free layer into the magnetic tunnel junction (MTJ), we demonstrate through micromagnetic simulations that near-continuous resistive modulation can be achieved based on the partially switched magnetic domains in the composite MTJ with a lateral dimension of 75 nm x 75 nm. A spin-orbit torque (SOT) MTJ with the proposed free layer achieves continuous modulations of MTJ conductance under input spikes, enabling a direct representation of the analog-valued membrane potential in the LIF neuron dynamics. Moreover, we observe from analyzing the micro-magnetic device simulations that the proposed spintronic neuronal device exhibits intriguing dynamics under spike excitation. In contrast to the standard LIF neuron model which has a constant leak time constant and a constant membrane resistance (defined as the sensitivity of membrane potential to input current magnitude), we find that the behavior of the proposed device can be characterized by a varying time constant and a varying membrane resistance. Specifically, both the membrane leak time constant and the membrane resistance are dependent on the transient membrane potential. Interestingly, we find that training SNNs for image classifications on the CiFAR-10 dataset^19^ using the device-inspired neuronal behavior achieves comparable test accuracy within fewer training epochs in comparison to training with the baseline neuron model. Moreover, we observe that the SNN incorporating the behavior of the proposed spintronic neurons demonstrates improved robustness under various types of noise injection, and such noise resiliency maintains under realistic device variations.

## Device and material fundamentals

### Spin-orbit torque magnetic tunnel junctions (SOT-MTJs)

**Figure 1:**
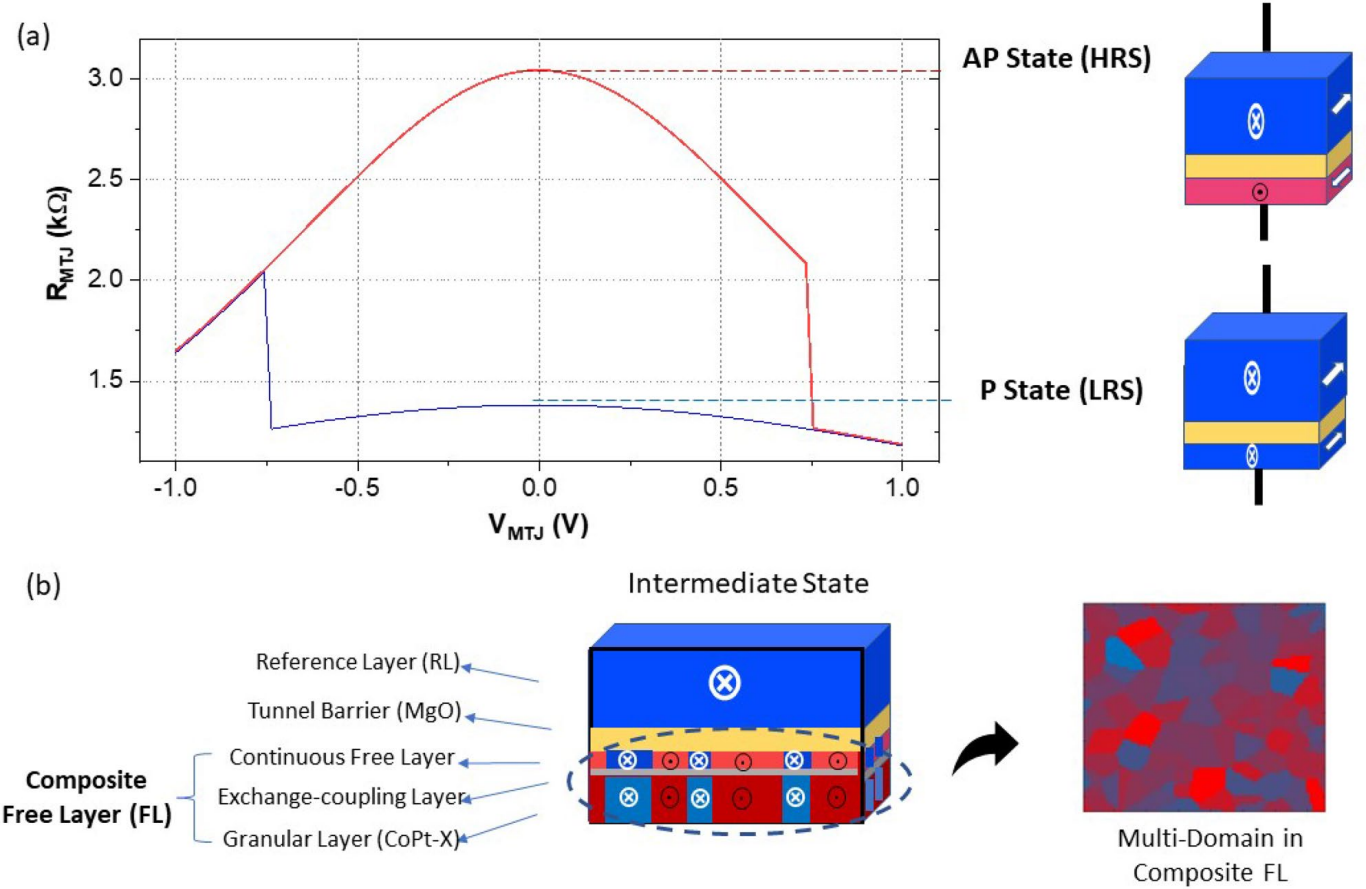
(**a**) High and low resistance states of an MTJ under voltage sweeps. The MTJ switches between parallel state (P) and anti-parallel (AP) state due to STT. The MTJ current–voltage characteristics are from device conductance modeling based on non-equilibrium Green’s function (NEGF) formalism^28^. (**b**) Device structure of the proposed MTJ with an exchange-coupled free layer. Multi-domain magnetization is induced by the granular layer in the composite free layer.

The proposed neuromorphic neuronal device is based on the structure of an MTJ, which has been developed for magnetic random access memory (MRAM). As is shown in Fig. 1, an MTJ is comprised of a tunnel barrier (MgO) sandwiched between two layers of ferromagnetic thin films—a reference layer (RL) with fixed magnetization and a free layer (FL) with changeable magnetic orientations. Conventionally, the FL of a fabricated device with critical dimension < 200 nm has a uniform magnetic ordering due to the combined effect of ferromagnetic exchange interactions and magnetic anisotropy, providing bi-stable MTJ resistance states following the relative orientation of the FL and RL being parallel (R_P_) or anti-parallel (R_AP_). The tunneling magneto-resistance (TMR) ratio, defined as TMR = $(R_{AP} -R_{P})/R_{P}$, quantifies the range of an MTJ’s resistance variation and will be utilized as the reading mechanism in our proposed device.

The magnetization of the FL in an MTJ can be switched by various types of stimuli such as external magnetic fields or spin (polarized) currents. Current-driven switching is preferred for scalable device technologies^20^. Charge current is spin-polarized as it goes through an MTJ, and the FL can be switched by the spin transfer torque (STT) induced by the spin-polarized current. Depending on the polarity of the current, the FL can be switched between the P and AP directions with respect to the RL^21^. Recently, it is found that in MTJ-heavy metal(HM) heterostructures, the FL can also be switched by spin-orbit torque (SOT) induced by a transverse electric current flowing in the adjacent heavy-metal layer^22^. SOT switching provides the opportunity of separate read and write paths, and eliminates the issue of applying large currents through tunnel barriers during STT write operations. Note that an SOT-MTJ requires a larger area than an STT-MTJ due to the 3-terminal configuration, and an extra transistor may be required in addition to the 1T/1R STT-MTJ cell^23^. But density requirement is less stringent for neuron activation compared to that for implementing synaptic weight storage, and SOT-MTJ may still achieve better density than CMOS-based implementations for emulating neurons.

### Multi-domain granular magnetic structure

In order to emulate the integration of membrane potential in a LIF neuron, it is desirable to provide reliable devices with continuous conductance modulation. In this work, we consider magnetic granular nanostructures for generating multiple states based on the switching dynamics of multi-domain magnetizations. The advances of magnetic multi-granular materials have been one of the major driving forces behind the recent progress of magnetic data storage in hard disk drive (HDD) industry, reaching an ultrahigh areal density of over terabytes per square inch (Tb/in^2^) with the averaged grain size below 10 nm^24, 25^. In a typical HDD granular storage medium, weakly coupled magnetic cores formed by Co-rich alloys are segregated by non-magnetic materials as grain boundaries. Since bits of information (“0” or “1”) are recorded as magnetic flux transitions across adjacent magnetic domains, granular boundaries play the critical role of reducing inter-granular exchange coupling and thus bringing down the magnetic cluster sizes. In the state-of-the-art multi-granular recording medium, it is observed that the domain widths in the down-track direction are reduced to 10–30 nm (in contrast to the domain size of 500–1000 nm in continuous ferromagnetic thin films^26^), providing a cost-effective mechanism for high-density data storage without involving complex processes of lithography. Note that granular materials with both in-plane and perpendicular magnetic anisotropy have been developed over the past decades. Depending on the material synthesis processes, magnetizations in the grains can be oriented following a uniaxial anisotropy direction with some distributions in the energy of magnetic anisotropy and the grain size^17, 25^.

By exploiting the nano-scale magnetic domains enabled by granular thin films, a multi-state MTJ can be made with a composite FL block based on exchange-coupled continuous/granular bilayer^27^. As is illustrated in Fig. 1, the continuous CoFeB layer of a regular MTJ is placed in proximity of a granular layer. The inter-layer magnetic coupling between the top continuous and bottom granular layers can be controlled by a thin non-magnetic exchange-coupling layer (typically made from Ru-rich alloy). The top continuous layer now serves as the sensing block to utilize the TMR reading mechanism, while the bottom granular layer serves as the block for information storage. In order to induce “fractured” magnetic domains, in addition to the binary “P” and “AP” configurations in the top block, a strong exchange-coupling is desired between the continuous and granular blocks. Therefore, when a varying fraction of the grains are aligned with the RL, intermediate magnetization states will emerge, leading to resistance modulation in such magneto-resistive devices. Although the usage of multi-granular systems has been widely applied for magnetic HDD industry, the potential of exploiting such scalable multi-domain magnetic structures for neuromorphic hardware is largely unexplored. In the following subsection, we will elaborate on the idea of leveraging the characteristics of magnetic granular nanostructures for building non-volatile spiking neurons.

## Non-volatile LIF neuron based on multi-state SOT-MTJ

### Multi-state SOT-MTJ

**Figure 2:**
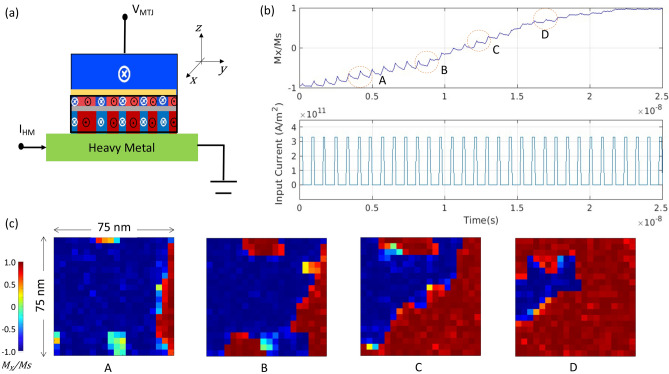
Mumax simulation of spin current driven multi-domain switching. (**a**) Device structure of a spin-orbit-torque driven MTJ with the exchange-coupled FL. (**b**) Evolution of the normalized M_X_ dynamics under input spikes. The spikes have a magnitude of 3.3e11 A/m^2^ with duration of 0.25 ns. (**c**) Snapshots of the magnetization during the progressive switching process. The transitions from blue to red represent the switching of local magnetic moments from M_X_/Ms = − 1 to M_X_/Ms = 1.

As is illustrated in Fig. 2, a multi-state SOT-MTJ is proposed exploiting the multi-domain magnetization in the composite FL. The sensing of the resistive states is done by monitoring the voltage across MTJ, utilizing the TMR mechanism. The MTJ conductance can be modeled by parallel conductance channels G_P_ and G_AP_, where the proportions of channels are determined by the magnetic domain configuration of intermediate states. As for write operation, we propose to use MTJ with SOT writing (SOT-MTJ) in our spin-based neurons for improved writing endurance and speed. Since it is desirable for neuronal hardware to have frequent and fast updates of the activation values, SOT-MTJ with the write current flowing through the HM layer, unlike a standard MTJ where the write current tunnels through the MgO, is preferred for endurance concerns. Depending on the duration and magnitude of input charge currents in the heavy metal layer (I_HM_), the composite FL can be partially switched. Conceptually, an inactive neuron has most of the device area unswitched, and a larger portion of the total device area being aligned to RL indicates that the neuron is closer to saturation under the input stimuli, as shown in the transition from point A to point D in Fig. 2. In the illustrated device, the magnetic granular structure has an in-plane uniaxial anistropy along the *X* direction. Note that both in-plane and perpendicular anisotropy can be exploited to construct an MTJ with exchange-coupled free layer. We choose to focus on in-plane anisotropy in the rest of the discussions, since deterministic SOT-switching of in-plane magnetization can be realized without the assistance of an external field or additional symmetry-breaking methods^29^.

We investigate the magnetization dynamics of the proposed multi-level device based on MuMax^3^ simulation^30^. MuMax^3^ is an open-source GPU-accelerated micromagnetic simulation program, where nano-scale temporal and spatial magnetization evolution is solved using a finite-difference discretization. Bilayer structures with lateral dimensions of 75–100 nm comprising the continuous cap layer and granular bottom layer are modeled. The thickness of the top layer is kept at 1 nm to make sure that the top layer can be fully controlled by the bottom layer, while the granular layer is set at 6–10 nm in order to provide sufficient thermal stability based on realistic magnetic anisotropy energy K_u_ = 0.4e6 J/m^3^. The inter-layer Ruderman–Kittel–Kasuya–Yosida (RKKY) exchange coupling coefficient is set to be strong between the top and bottom (see “Methods” for more details). Granular configurations with an average grain size of 8–10 nm are introduced by utilizing the built-in extension of Voronoi tessellation in MuMax. The variance of magnetic properties among grains is introduced by adding a Gaussian distribution to the Ku at initialization, approximating a 20–25% switching field distribution as observed in experiments on magnetic granular structures. Further details can be found in “Methods” Section.

As shown in Fig. 2b The normalized magnetization shows gradual switching from completely AP (normalized M_x_ = − 1) to P (normalized M_x_ = 1) over time under repetitive pulses of input current. The observed sloped switching of the magnetization, instead of a step-like reversal, validates the feasibility of utilizing the magnetic switching to represent a continuously varying membrane potential. Different mechanisms can contribute to the observed gradual magnetization switching. First, the stochastic nature of magnetization dynamics under thermal agitation could lead to the non-uniform onset of switching of individual grains under the input currents. Moreover, our simulations include distributions of switching energy as well as the role of magneto-static interactions among the grains. It is found that reproducible non-coherent partial switching can be achieved when monitoring an ensemble of more than 50 nano-grains, corresponding to a lateral dimension of 75 nm with the averaged grain size of 8–10 nm.

Noticeably, during the process of switching under current pulses, the total magnetization always shows some decaying at the end of each pulse. The decay is caused by the switching of local magnetic moments under the combined influence of thermal agitation, inter-granular exchange coupling, and the demagnetizing field due to magnetic dipole interaction. When the input pulse is gone, some of the switched grains may flip due to the combined force of thermal attempt and local dipole interactions. As discussed further in the following section, the observed decay of magnetic signal can be exploited to imitate the leak effect for a LIF neuron model. Note that most of the decay in magnetization occurs within the first few nanoseconds after input pulses, and the intermediate states with partially switched domains stabilize, thanks to sufficient average thermal stability of the magnetic grains. The stability of intermediate states enables non-volatility in these analog neuronal devices, potentially leading to high energy efficiency by reducing the need to offload the intermediate neuronal states to off-chip memory.

### LIF non-volatile neuron circuit block and firing dynamics

The key components in hardware emulation of the LIF neuronal dynamics are membrane potential and leak. We use magneto-resistive states of the analog MTJ to represent a (normalized) membrane potential, which can be linearly mapped to the magnetization M_x_ (ranged between -M_s_ and M_s_). The integration process of membrane potential can be emulated by the gradual non-coherent multi-domain magnetization switching demonstrated in the earlier section. As for the leak, we rely on the observed relaxation of local magnetic moments under the influence of thermal agitation and dipole interaction. The LIF neuron model can now be mathematically expressed as the following:

1 $$\begin{aligned} \frac{dV_{mem}(t)}{dt}&= \frac{1}{\tau } \left( -\left( V_{mem} -V_{rest}\right) +R_{mem}\cdot I\right) \end{aligned}$$

2 $$\begin{aligned} V_{mem}&= \frac{{}M_{x}/M_{s} +1}{2} \end{aligned}$$

where $V_{mem}$ is the membrane potential, *I* represents the input current obtained from the weighted summation of spiking inputs from pre-neurons, $\tau$ denotes the time constant for membrane potential leak, and $R_{mem}$ is the effective neuron membrane resistance which characterizes the sensitivity of membrane potential to input currents. $V_{rest}$ is resting potential and set to zero throughout our simulation.

**Figure 3:**
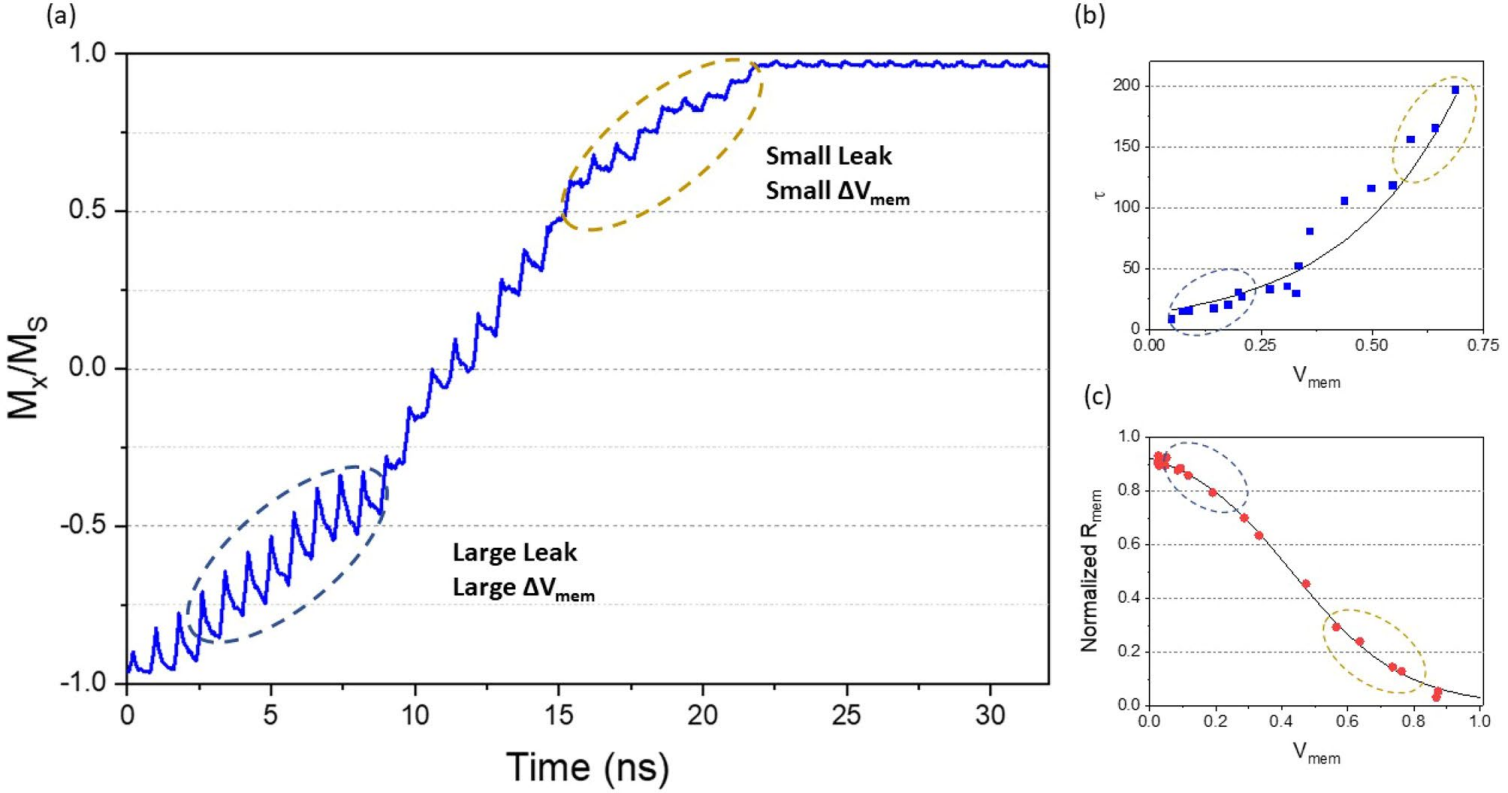
Detailed analysis of the dynamic behavior of the simulated device. (**a**) The magnetization versus time under the stimulus of input spike sequence. (**b**) Leak time constant $\tau$ versus the instantaneous $V_{mem}$. Data points (blue squares) are extracted from the plot in (**a**), and numerically fitted by Eq. (3). (**c**) Normalized membrane resistance $R_{mem}$ versus the instantaneous $V_{mem}$. Data points (red dots) are extracted from the plot in (**a**), and numerically fitted by Eq. (4).

We first investigate the simulated device dynamics under the excitation of input currents in more detail. As shown in Fig. 3a, the membrane potential $V_{mem}$ grows gradually from 0 to 1 under evenly spaced spikes of identical magnitudes (similar to the case of Fig. 2). The membrane potential grows when input spikes are present, and decays (leaks) during the intervals between spikes. Interesting detailed features can be observed from the membrane potential versus time curve in Fig. 3a. Specifically, the “peaks” in $V_{mem}$ are sharper at the initial stage when $V_{mem}$ is small, while the whole curve becomes less spiky as $V_{mem}$ gradually grows towards saturation. Mathematically such modulation of $V_{mem}$ can be described by a combination of an increasing leaky-time constant $\tau$, and a decreasing differential membrane resistance $R_{mem}$. The highlighted low (high) $V_{mem}$ regimes in Fig. 3a correspond to large (small) leak and large (small) differential resistance. We further find that the dependence of decay time constant $\tau$ on the $V_{mem}$ as shown in the micromagnetic simulation approximately follows an exponential increase, as is illustrated in Fig. 3b. Such leaky behavior can be contributed to the interaction of the exchange-coupled multi-granular system. Intuitively the exchange coupling among grains tends to form ferromagnetic clusters among neighbors and inhibit partial switching at the initial stage of excitation. Moreover, it is also found that the membrane potential $R_{mem}$, which represents the sensitivity of $V_{mem}$ to input current, decreases as the initial $V_{mem}$ increases (see Fig. 3c). The varying $R_{mem}$ is also originated from the interactions among grains with switching field distribution. Such reduction of sensitivity is conceptually similar to the phenomena of fatigue and homeostasis observed in biological neurons. We incorporate the observed varying $\tau$ and varying $R_{mem}$ into the LIF neuron behavior model based on numerical fitting of the micromagnetic dynamics in the following:

3 $$\begin{aligned} \tau&= \tau _0 exp\left( k\cdot V_{mem}\right) \end{aligned}$$

4 $$\begin{aligned} R_{mem}&= \frac{R_0}{exp(a\cdot V_{mem}-b)+1} \end{aligned}$$

where $\tau _0$ and $R_{0}$ represent the initial leak time constant and membrane channel resistance respectively, and other parameters are numerical fitting parameters. Based on the device simulation and numerical fitting, a compact behavior model with details including the $V_{mem}$-dependent $\tau$ and $R_{mem}$ is constructed and validated for reproducing the functionality of membrane potential accumulation and spike firing.

**Figure 4:**
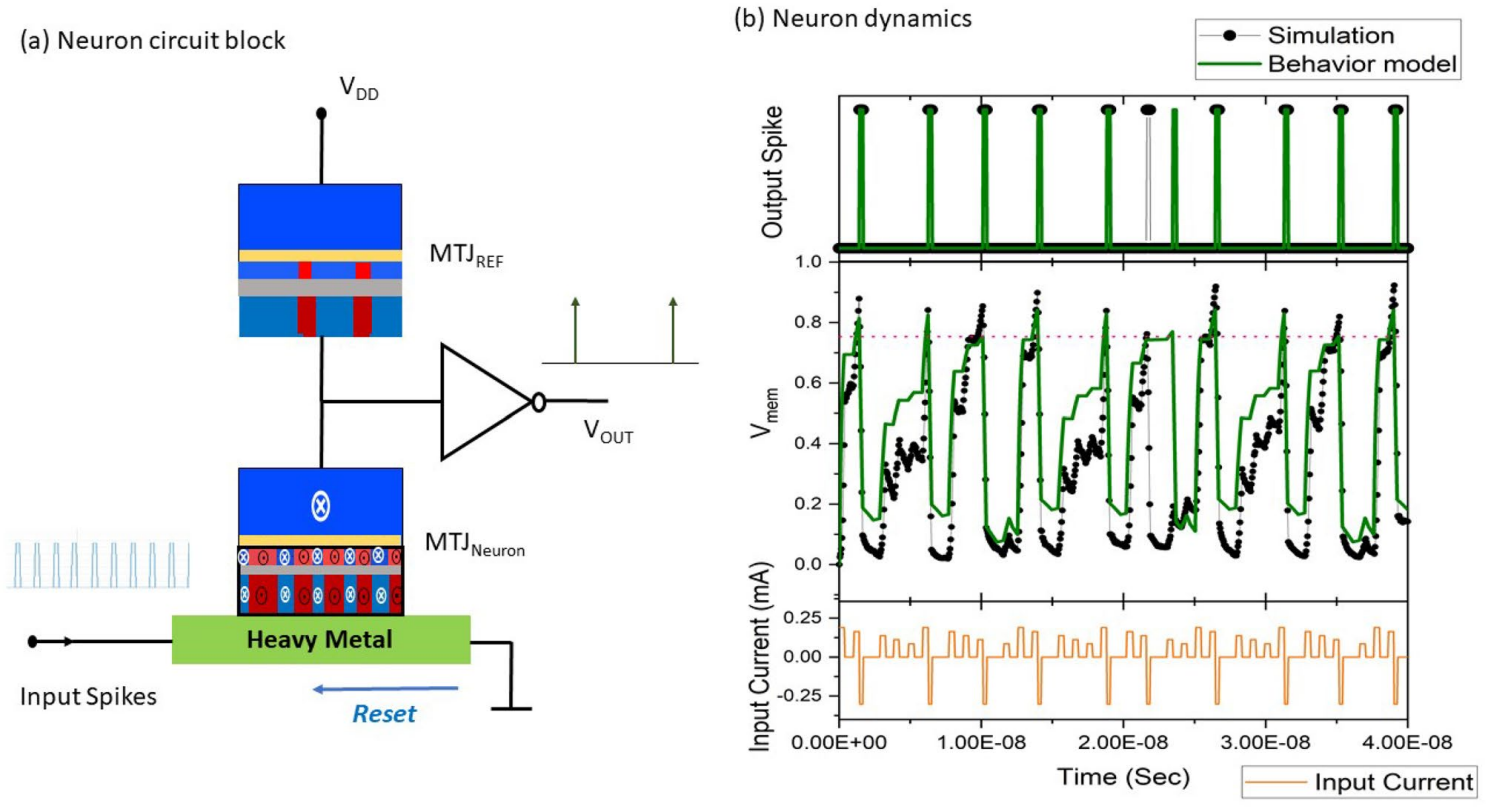
(**a**) The spiking neuron circuit block incorporating the proposed multi-domain SOT-MTJ. (**b**) Demonstration of leaky integrate and fire dynamics in micro-magnetic simulation. The spiking dynamics is also reproduced by the behavior model. The firing threshold of $V_{mem}$ is set at 0.75.

Leveraging the multi-domain device dynamics, an exemplary circuit implementation of SOT-MTJ spiking neuron is proposed and illustrated in Fig. 4. The neuron MTJ connected in series with a reference MTJ is placed on top of a heavy metal layer. Input spikes are supplied as electrical currents flowing in the HM layer to drive the switching of the adjacent MTJ_Neuron_. Reference MTJ is set to a fixed configuration and remains unchanged while the neuron device is being excited. As an increasing portion of magnetic domains in the FL of MTJ_Neuron_ are switched under input currents, the voltage across the MTJ_Neuron_ changes proportionately. The varying voltage over MTJ_Neuron_ is connected as an input to the inverter, forming a resistive divider network. Resetting of the SOT-MTJ neuron is done by applying currents with the opposite polarity of the input spikes. It has been demonstrated that the timing of iterative read/write and reset can be achieved by incorporating only 2–3 access transistors, leading to a compact design for generating spiking output based on such resistive divider circuitry^13, 31^. Based on the proposed device and circuit, we simulate the magnetization dynamics of the multi-state MTJ with the inclusion of the firing and reset operations. We build a behavior model to emulate the magnetization dynamics, and validate the neuronal behavior model by reproducing the firing dynamics obtained from the micromagnetic simulation. In the validation, we consider input currents with varying amplitudes to reflect the scenario that neurons in a multi-layer SNN will receive weighted input spikes of varying magnitudes. As is shown in Fig. 4, the behavior model (green line) approximately follows the trace of the micro-magnetic simulation (black dots), and both of simulation and behavior model demonstrate $V_{mem}$ accumulation leading to output spikes. The comparison also shows that the spiking rate averaged over extended time steps is quantitatively captured by the behavior model (although some small deviation in the timing of spikes may occur due to the error of the behavior model combined with inherent stochasticity in magnetization switching).

**Figure 5:**
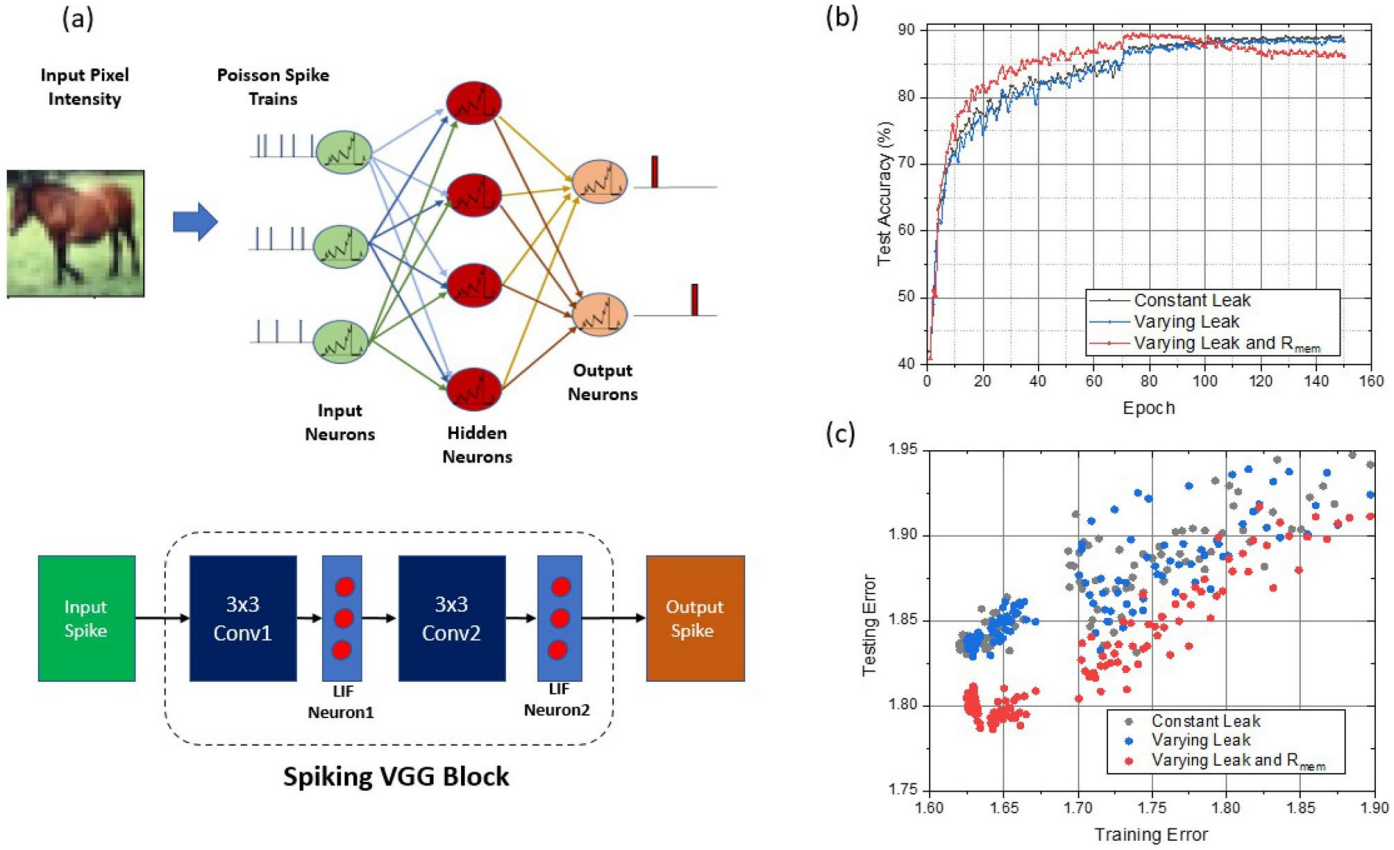
(**a**) Illustration of the SNN architecture of VGG-type convolutional neural network. (**b**) Test accuracy versus training epoch for the SNNs using various neuron models. (**c**) Plot of test error versus training error for the SNNs using various neuron models.

The above simulations demonstrate that the functionality of LIF neurons can be realized directly in the proposed analog SOT-MTJ devices in a very compact fashion. In the following, we discuss the achieved energy efficiency of the proposed spiking spintronic neuron in comparison with CMOS neurons. Since the write and reset of the MTJ_Neuron_ is done by applying the HM metal, we can estimate the device-level energy consumption by calculating $I^{2}*R_{HM}*t_{WR}$. Based on the device parameters used in the micromagnetic simulations, we can obtain a charge current on the order of 25–61 μA with spin-charge conversion efficiency $\Theta _{SH}$= 0.3 and R_HM_ = 830 $\Omega$ using Ta as the HM layer. The energy per spike generation following the device dynamics as illustrated in Fig. 4 is estimated to be 0.22 pJ based on summing the energy consumed over integrated time steps (0.16 pJ) and the reset energy (0.06 pJ). In contrast, the state-of-the-art design of CMOS neurons is reported to have energy consumption at 41.3 pJ^8^ while requiring a large number of transistors. Moreover, the proposed spin-based device provides non-volatile analog states, which could lead to significant energy saving by eliminating the need of off-chip memory access to store and load the intermediate activation states throughout the accumulation and update of membrane potential in the neuron. The energy and area advantages offered by the spin-based non-volatile spiking neuron can potentially lead to massive improvement in the computational efficiency of neuromorphic hardware.

## Improved noise resiliency

**Figure 6:**
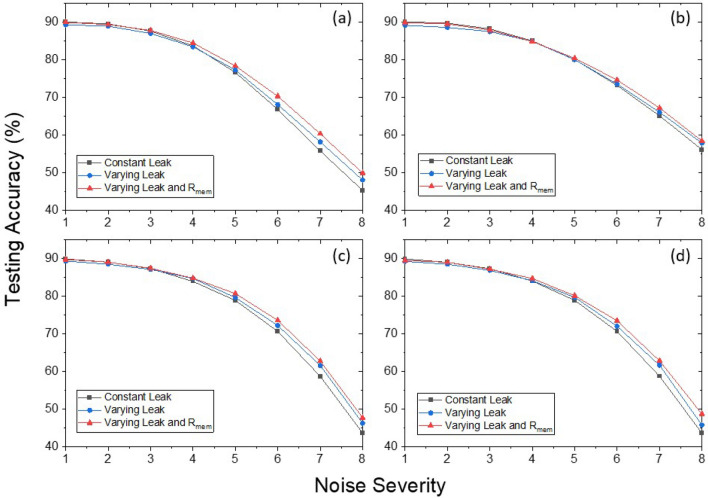
Testing accuracy vs noise severity Spiking VGG9 on Cifar10. (**a**) Gaussian noise injected to input before Poisson spiking train generation (scenario-1). (**b**) Gaussian noise injected after Poisson spiking train generation (scenario-2). (**c**) Impulse noise injected to input before Poisson spiking train generation (scenario-1). (**d**) Impulse noise injected after Poisson spiking train generation (scenario-2)

In this section, we will explore the algorithm-level performance of the proposed neuron when deployed in a deep spiking neural network. The behavior model illustrated in Fig. 4 is incorporated into large spiking neural network architecture for image classification. An exemplary SNN architecture is illustrated in Fig. 5a, where the pixel intensity of input images is converted to Poisson spike trains and fed into a multi-layer neural network composed of spiking neurons. We leverage the recent development on the training of convolutional SNN as presented in recent literature^32^. In our approach, spike-based backpropagation algorithm is enabled for training deep SNNs with LIF neurons following an approximate derivative method that accounts for the leaky behavior of LIF neurons.

We start with the baseline spiking VGG9 model with regular LIF neurons having a constant leak rate $\tau$=100, and then retrain two modified SNN models where the conventional LIF neurons are replaced by the modified neurons after each convolution layer. Both varying leaking rates ($\tau$) and the varying sensitivity of membrane potential to input currents ($R_{mem}$) are incorporated in the behavior neuron model in the modified SNN. The parameters used in the baseline LIF neuron model have shown satisfactory results^7^ for image classifications on CiFAR-10 dataset, providing a good reference starting point for the algorithm-level demonstration. Details such as the setting of hyper-parameters for training are included in “Methods” Section. We first analyze the impact of introducing only the varying leak $\tau$ into the neurons, and then further investigate the total effect of having both a varying $\tau$ and a varying membrane resistance $R_{mem}$. As is shown in Fig. 5b, all models trained with modified LIF neurons can reach an accuracy of 89–90% on Cifar-10 dataset after training. This observation confirms that it is feasible to reach near-ideal test accuracy when realistic device characteristics of the proposed SOT-MTJ are taken into account. Particularly, the neuron model that captures the device characteristics most accurately by including both varying $\tau$ and varying $R_{mem}$, achieves the best test accuracy with the smallest number of epochs needed. Moreover, we also plot the trend of testing error versus training error. Conceptually, reaching a lower testing error at the same training error implies superior algorithmic capability, which can contribute to faster convergence and better generalization. As is shown in Fig. 5c, a trend of the data cloud shifting towards the bottom right indicates that the modified proposed device-inspired neuron model can help to reduce the testing error at a fixed training error. Such reduction in testing error reflects the potential of improvement using neuron models with varying $\tau$ and $R_{mem}$. We will demonstrate the impact of the introduced neuron characteristics on boosting the algorithm-level noise resiliency.

**Figure 7:**
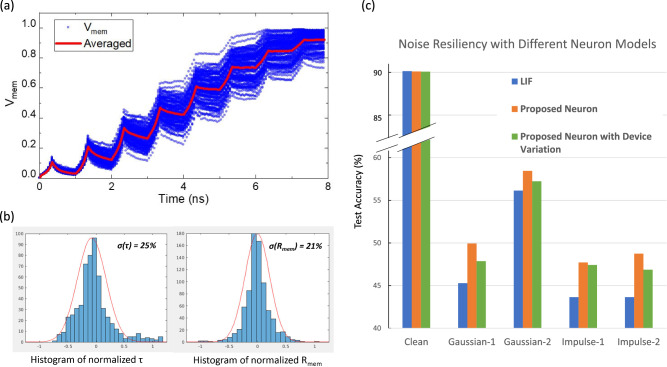
Noise Resiliency of Neuron Models with non-ideal device variations. (**a**) Traces of membrane potential accumulation under input spikes for 100 runs with different initialization of the multi-granular configuration. (**b**) Statistics of the model parameters under device variation. (**c**) Summary of SNN model performance in presence of device variation in comparisons with baseline models

In the following noise experiments, we explicitly show that the neuron models with varying $\tau$ and $R_{mem}$ provide remarkable improvement in resiliency against various types of noises. We evaluate the model with varying $\tau$ only, and the model with both varying both $\tau$ and $R_{mem}$, in comparison with the baseline model using constant leak LIF neurons with $\tau$=100. We run experiments to see the capability of maintaining a certain prediction accuracy under stochastic perturbations to the input of deep SNN. Two types of random noises, Gaussian noise and impulse noise^33^, are considered. For each type of noise pattern, we look into two scenarios of noise injection to the input^7^. In scenario-1, random noise is added to image pixels at each time step before the generation of Poisson spike trains, which leads to possible modification of the timing and spiking frequency of spike trains, while the resultant spikes are still binary. In scenario-2, random noise is added at each time step to the already generated spike trains so that the magnitude of spikes is no longer binary due to the addition of noises, although the timing of the spike trains is intact. As is shown in Fig. 6, accuracy degradation with increasing noise severity defined by the averaged magnitude of the injected noise is observed for all types of noise perturbations. Moreover, with a similar clean testing accuracy for the models with different neuronal dynamics, the noise-injecting experiments clearly show that SNNs with $V_{mem}$-dependent $\tau$ and $R_{mem}$ (blue, red) achieve improved robustness. Across all 4 scenarios of noise perturbations, LIF neurons with both $\tau$ and $R_{mem}$ being $V_{mem}$-dependent perform the best, showing about 5% improvement at the maximal noise severity compared to the baseline model. Intuitively, the varying leak behavior in our proposed devices exhibits faster leak (small $\tau$) at the initial stage of switching, which could help to filter out small input noises added to inputs and only have substantial accumulations of $V_{mem}$ when input is strong enough. On the other hand, the $R_{mem}$ reduces as $V_{mem}$ accumulates, which indicates that the neurons have growing fatigue under further stimuli. Such reduction in sensitivity resembles a homeostasis process and prevents the stimulated neuron from generating output spikes too frequently. As is illustrated in Fig. 5c, incorporating the varying $R_{mem}$ significantly shifted down the testing error at the same training error, indicating a model of stronger generalization capability and less overfitting of the training dataset. It is worthwhile to mention that the baseline LIF neurons with constant $\tau$ have already been demonstrated to have improved noise resiliency compared to IF neurons without leak^7^. Our observation demonstrates that incorporating the more complex leaking behavior and membrane potential sensitivity inspired by the characteristics of multi-domain spin devices can further enhance the robustness of spiking neurons against noisy inputs. Note that the tested SNN models are all trained with clean input and have no prior knowledge of the various types of noise injection, and yet the improvement in noise resiliency is clearly observed for all the scenarios. Therefore, implementation of the proposed spintronic analog neuron provides an efficient pathway for gaining robustness from the underlying device technology in hardware implementation, in contrast with the approach of retraining with various types of noisy perturbation, which typically consumes significant additional computational resources.

As with all kinds of analog devices, noises associated with device variability can be a concern for practical implementations. We demonstrate the impact of realistic device variation on the performance of deep SNNs incorporating the proposed spintronic neuronal devices. The major contribution to the device variation in this multi-granular MTJ-based design is the stochasticity of the switching of magnetic moments in combination with the switching field distribution among the grains. Fig. 7a illustrates 100 simulated traces of the $V_{mem}$ dynamics of the proposed multi-domain devices with different random initialization. As is shown in Fig. 7b, we observe clear variations in both $\tau$ and $R_{mem}$ for the given device specification (lateral dimension of 75 nm × 75 nm, with an averaged grain size of 8 nm and switching field distribution of 25% among the grains). Subsequently, we incorporate the observed variation as Gaussian noise to the neuron parameters of the behavior model, and evaluate the SNN inference accuracy in presence of such non-ideality. We find that the clean SNN inference accuracy based on the non-ideal neurons sees negligible degradation compared to references, corroborating the improved robustness of the proposed neuron. As is summarized in Fig. 7c, under the noise injections, the test accuracy with the non-ideal neurons shows slight drops compared with the performance of ideal models, but the model with non-ideal neurons still outperforms the baseline SNN with simple LIF neurons. Our demonstration confirms the feasibility of implementing the proposed spin-based neuron device under practical device variability in hardware implementation.

## Conclusion

We propose a novel spintronic neuro-mimetic device that can emulate the leaky integrate-and-fire neuron dynamics with high energy efficiency and compact footprints. Based on the proposed free layer with continuous-granular composite structure, we achieve gradual switching of the magnetization, which is utilized to emulate both accumulation and leak of membrane potential. Furthermore, we observe unique characteristics of a varying leak rate and a varying membrane channel resistance from the micromagnetic simulations of the proposed device. After incorporating the observed device behavior into the spiking neuron models, we demonstrate improved robustness against various types of input noise injection for image classifications. The proposed analog magneto-resistive devices leveraging memory and storage technologies could open up exciting new avenues for developing emerging neuromorphic hardware. Moreover, our cross-layer exploration suggests that the tuning of the leak and membrane resistance of spiking neurons may lead to an interesting pathway towards efficient and robust bio-plausible learning algorithms.

## Methods

### MuMax simulation details

The magnetization dynamics of composite free layer is simulated in MuMax3 as a bilayer magnetic structure of distinctive magnetic properties in each layer. The top continuous capping layer represents CoFeB, which is most commonly used in MTJ stacks. The capping layer is set as a soft ferromagnetic layer with negligible magneto-crystalline anisotropy and strong lateral exchange coupling. The bottom granular layer setting is based on Cobalt-based granular magnetic medium utilized in mass production of magnetic hard disk drives. Voronoii tessellation as provided by MuMax is used to generate initial granular configuration following a specified random seed. Considering the segregation of non-magnetic materials in granular structures, inter-granular coupling is reduced to 1–10% of the coupling strength in a continuous film. Such reduced inter-granular coupling will enable the occurrence of multi-domain intermediate states during magnetic switching. For the specific data analyzed in the paper, the Gilbert damping constant is set at 0.01 for both top and bottom layers. The continuous top layer has large saturation magnetization Ms_top_ = 400e3 A/m with large exchange coupling Aex = 1e–11 J/m, while the granular layer has lower magnetiztion Ms_bottom_ = 250e3 A/m with inter-granular coupling of 1e–13 J/m. The grains in the bottom layer have an averaged in-plane uni-axial magnetic anisotropy of K_u_ = 0.4 e6 J/m^3^ with 20–25% grain-to-grain variation as is observed in actual storage medium. The thicknesses of the top and bottom layers are 1 and 6 nm, respectively, so that grains in the bottom layer can reach a thermal stability factor KuV/kT = 37 at room temperature for grain size = 9 nm and thus maintain thermally stable during the sub-μs neuron dynamics. The inter-layer RKKY exchange coupling is set to be as high as 50% of the lateral exchange coupling (A_RKKY_ = 5e–12 J/m) to ensure a strong coupling so that local magnetic moments from the top layer will follow the local magnetization in the grains of the bottom layer.

### SNN training

The SNN models are trained with clean data following the approach of spike-based back-propagation as proposed in^32^ with a batch size of 16–32. Each model is trained with 150 epochs, where the learning rate reduces at the 70th and 100th epochs. Input encoding to Poisson spike trains of 100 time steps is done after normalization of pixel intensity of input images to the range of [–1, 1] with a mean of 0. For evaluations of those device-based neuron models, the last fully-connected layer uses regular LIF neuron, as it is observed in our experiment that when neurons with varying leakage are used, the last layer of SNN models is required to have LIF neurons with small leak ($\tau$ $\sim$ 100) in order to reach convergence. The VGG-based multi-layer spiking model is constructed using Pytorch.
